# Antimycobacterial Activity: A New Pharmacological Target for Conotoxins Found in the First Reported Conotoxin from *Conasprella ximenes*

**DOI:** 10.3390/toxins10020051

**Published:** 2018-01-23

**Authors:** Andrea Figueroa-Montiel, Johanna Bernáldez, Samanta Jiménez, Beatrix Ueberhide, Luis Javier González, Alexei Licea-Navarro

**Affiliations:** 1 Departamento de Innovación Biomédica, CICESE, Carretera Ensenada-Tijuana 3918, Ensenada, BC C.P. 22860, Mexico; figuerm@cicese.edu.mx (A.F.-M.); jbernald@cicese.edu.mx (J.B.); mjimenez@cicese.edu.mx (S.J.); 2 Department of Biochemistry and Molecular Pharmacology, New York University, Langone Medical Center. 430 East, 29th Street, New York, NY 10016, USA; beatrix.ueberheide@nyumc.org; 3 Laboratorio de Espectrometría de Masas, Departamento de Proteómica, Centro de Ingeniería Genética y Biotecnología, Avenida 31 e/158 y 190, Cubanacán, Playa, P.O. Box 6162, C.P. 10600 La Habana, Cuba; luis.javier@cigb.edu.cu

**Keywords:** *Conasprella ximenes*, mass spectrometry, EThcD, transcriptome, conotoxins, de novo sequencing, antimycobacterial, tuberculosis, The first conotoxin reported for *C. ximenes* with anti Mycobacterium activity and suggest that other venoms may also contain biological active principles for this new pharmacological target not explored before for Conidae species.

## Abstract

*Mycobacterium tuberculosis* is the etiological agent of tuberculosis, an airborne infectious disease that is a leading cause of human morbidity and mortality worldwide. We report here the first conotoxin that is able to inhibit the growth of *M. tuberculosis* at a concentration similar to that of two other drugs that are currently used in clinics. Furthermore, it is also the first conopeptide that has been isolated from the venom of *Conasprella ximenes.* The venom gland transcriptome of *C. ximenes* was sequenced to construct a database with 24,284 non-redundant transcripts. The conopeptide was purified from the venom using reverse phase high performance liquid chromatography (RP-HPLC) and was analyzed using electrospray ionization-mass spectrometry (ESI-MS/MS). No automatic identification above the identity threshold with 1% of the false discovery rate was obtained; however, a 10-amino-acid sequence tag, manually extracted from the MS/MS spectra, allowed for the identification of a conotoxin in the transcriptome database. Electron transfer higher energy collision dissociation (EThcD) fragmentation of the native conotoxin confirmed the N-terminal sequence (1–14), while LC-MS/MS analysis of the tryptic digest of the reduced and S-alkylated conotoxin confirmed the C-terminal region (15–36). The expected and experimental molecular masses corresponded, within sub-ppm mass error. The 37-mer peptide (MW 4109.69 Da), containing eight cysteine residues, was named I1_xm11a, according to the current nomenclature for this type of molecule.

## 1. Introduction

*Mycobacterium tuberculosis* (Mtb) is the etiological agent of tuberculosis (TB), an airborne infectious disease that is a leading cause of human morbidity and mortality worldwide. In spite of the development of new strategies to deal with this highly prevalent pathogen, according to the World Health Organization, there were 10.4 million new cases of TB, and 1.4 million deaths related to TB, in 2015 [1]. Nowadays, front-line treatment of TB combines four drugs (rifampicin, isoniazid (INH), pyrazinamide, and ethambutol (EMB)), administered over six to eight months. Consequently, efforts in the development of new pharmaceuticals are focused on molecules with new targets, to shorten and improve treatment outcomes. The growth and nutritional requirements of mycobacteria have been studied intensely; however, as with all bacteria, nutrient uptake mechanisms depend on the permeability barriers imposed by the cell envelope. For this reason, these pathways have been considered possible molecular targets to destabilize and inhibit the growth of mycobacteria [2].

Several studies have reported that marine peptides exhibit various anti-infective activities, such as antimicrobial, antifungal, antimalarial, and anti-mycobacterial activities [3]. There has been an increasing realization that venom is a large source of chemical diversity that could be successfully used in the pharmaceutical industry [4]. Within venomous species, marine gastropods of the Conidae family are known for the hyperdiverse array of toxins they produce, which are commonly referred to as conotoxins or conopeptides. These molecules are well-known as being highly selective to their molecular targets, which include the receptors or ion channels of cell membranes [5], making them great candidates for deterring bacteria, such as mycobacteria. Each of the 800 species of cone snails discovered to date generates around 1000 different venom peptides [6], and only ~5% overlap between species [7]. From the total number of conopeptides, only a small fraction (~0.1%) have been pharmacologically characterized [4], yielding numerous choices of untested conopeptides to prove in the medical field. Thus far, the antimycobacterial properties of peptides derived from Conidae species have not been reported on.

Using liquid chromatography, Conidae venom can be fractionated and screened for important biological functions. However, a key step between the screening of a conotoxin and the identification of its molecular targets, is undoubtedly determining knowledge regarding its primary structure—a task that presents several challenges. Not only is cone snail venom extremely complex, but conotoxins are also small and well-structured peptides, consisting of 10 to 50 amino acid residues, with several cysteine residues linked by multiple disulfide bonds and a high number of unusual post-translational modifications (PTMs) [8].

Mass spectrometry has emerged as an important analytical technique for conotoxin characterization, due to its high sensitivity and throughput analyses. In particular, the ability to perform liquid chromatography, coupled with tandem mass spectrometry (LC-MS/MS) analysis, is a determinant for successful de novo sequencing, conotoxin identification in sequence databases, and PTM characterization [9].

De novo sequencing of conopeptides using mass spectrometry represents a challenge because their molecular masses often exceed the optimum fragmentation size using collision induced dissociation (CID) [10,11]. Additionally, conopeptides frequently contain several basic amino acids that limit the availability of mobile protons, representing an obstacle for efficient fragmentation with CID [12]. Rather than obtaining a full sequence using mass spectrometry, it seems more realistic to obtain partial sequences, or sequence tags, to allow identification of toxins in sequence databases. Some of these limitations can be overcome by using other complementary fragmentation techniques, based on different principles, such as electron transfer dissociation (ETD) [13,14,15], or electron transfer higher energy collision dissociation (EThcD), a hybrid fragmentation technique [16,17] that possesses the merits of two fragmentation techniques, ETD and higher-energy C-trap dissociation (HCD) [18,19].

However, the lack of available cDNA databases for most of these organisms imposes a serious limitation in the identification of conopeptides using mass spectrometry [20]. Protein identification and de novo sequencing are not mutually-exclusive strategies [21], and can be combined to identify a conopeptide of interest by extracting a reliable sequence tag from the MS/MS spectra [22].

The transcriptome encloses all genes that are being actively transcribed in a specific organ [23]. In cone snails, the production of conotoxins is limited to the venom gland, commonly known as a venom duct. Therefore, in order to construct a database that contains the conotoxins of a particular cone snail, sequencing the venom duct transcriptome is ideal.

*Conasprella ximenes* is a cone snail present in the coastal region of the Sea of Cortez (Mexico) and its conopeptides have never before been studied. In this work, we screened *C. ximenes* venom to assess the presence of antimycobacterial activity. The venom gland transcriptome of this species was sequenced and assembled to facilitate the identification of conotoxins in the LC-MS/MS experiments. As a result, the first conotoxin reported for *C. ximenes* was named I1_xm11a and showed activity against Mtb in the low-micromolar range.

## 2. Results

### 2.1. Venom Fractionation for Anti-Mtb Activity Evaluation

**Figure 1:**
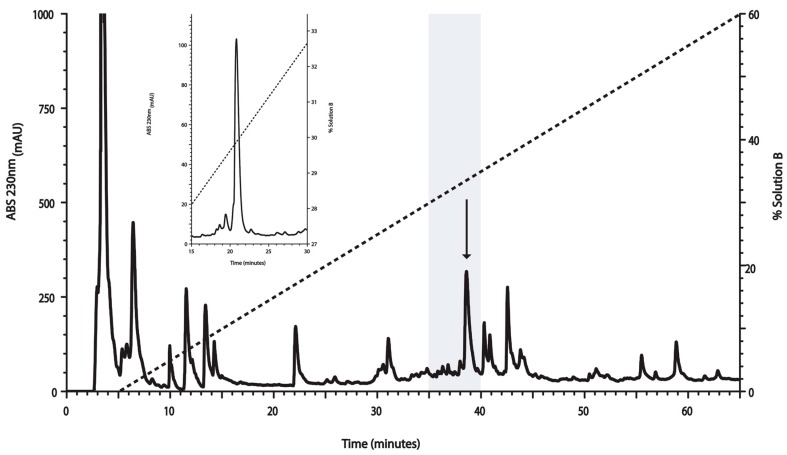
Fractionation using RP-HPLC of *Conasprella ximenes* venom on a linear gradient, from 0% to 60% of Solution B. The gray area represents the fraction with activity against *Mycobacterium tuberculosis* (Mtb) and the arrow indicates the elution time for the conotoxin of interest (I1_xm11a). This fraction was further repurified (inset) with a slower linear gradient, from 22% to 45% of Solution B (only part of the chromatogram is shown). In both RP-HPLC runs, the broken lines indicate the linear gradient of Solution B. This peak was evaluated in further biological assays to determine its minimal inhibitory concentration, and was also characterized using mass spectrometry to reveal its chemical identity (Section 2.4, Section 2.5 and Section 2.6).

The RP-HPLC profile of the crude venom extract from *C. ximenes* is shown in Figure 1. Based on the pharmacological backgrounds of conotoxins used for diverse biomedical approaches [24], the venom components from *C. ximenes* were collected in pools every five minutes. This led to 13 fractions, which were screened to evaluate their antimycobacterial potential (see Section 2.2), which resulted in a fraction (highlighted in gray in Figure 1) with the desired biological activity. This fraction was further fractionated using RP-HPLC, using a slower gradient (see inset of Figure 1), and the biological activity of this fraction remained in the peak, indicated by an arrow (Figure 1).

### 2.2. Antimycobacterial Susceptibility Assay

**Figure 2:**
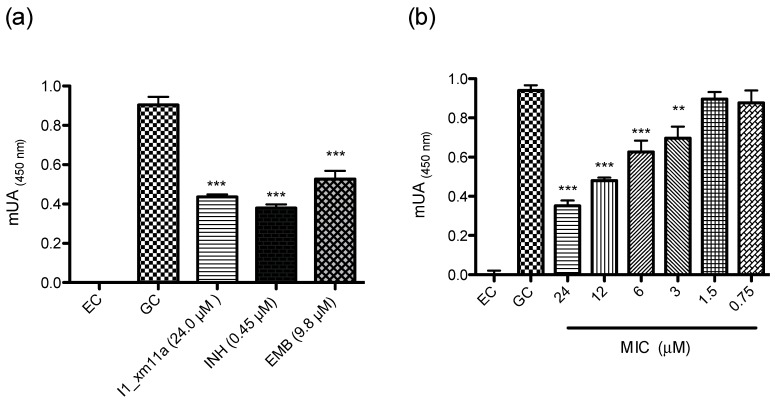
(**a**) Growth-inhibitory effect of I1_xm11a peptide against pathogenic *M. tuberculosis* (H37Rv strain). Minimal inhibitory concentration (MIC) cut-off values for positive controls, ethambutol (EMB) and isoniazid (INH), were 9.8 and 0.45 µM, respectively (Figure S1). The experimental control (EC) corresponds to sterility media without inoculum; (**b**) The MIC range at 24–0.75 µM of conotoxin was evaluated. The statistical significance of differences between treatments and growth control were analyzed using a Student’s *t*-test. ** *p* < 0.01, *** *p* < 0.001 vs. Growth Control (GC).

The pure peptide was tested at 100 µg/mL (24 µM) in an antimycobacterial susceptibility assay on the H37Rv strain (Figure 2a); activity was confirmed at a micromolar concentration. Subsequently, the minimal inhibitory concentration (MIC) of the peptide was evaluated at a concentration range of 24–0.75 µM (Figure 2b), and was determined at 3 µM.

### 2.3. Venom Gland Transcriptome Sequence Database

To facilitate the identification of the conotoxin of interest via mass spectrometry using a proteomic approach, the transcriptome of the venom gland was sequenced. RNA was extracted from a venom gland pool of *C. ximenes* (168 μg/mL), and, after assessing the quality and quantity, was considered adequate for sequencing. RNA-seq produced a total of 56,363,079 good-quality reads (confirmed by means of FastQC). After translating them into proteins (longer than 50 amino acids), and removing redundant information, a sequence database comprising 24,284 total sequences was constructed.

### 2.4. ESI-MS Analysis of the Native and Carbamidomethylated Peptide

**Figure 3:**
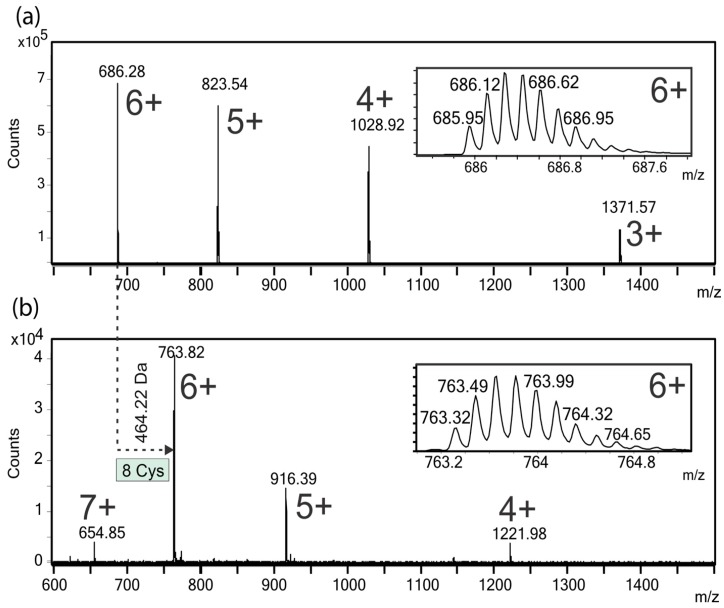
Electrospray ionization-mass spectrometry (ESI-MS) spectra of the native (**a**) and reduced S-carbamidomethylated; (**b**) conotoxin, I1_xm11a. In both mass spectra, the insets show the isotopic ion distributions of the corresponding [M + 6H]^6+^ ions. The molecular mass difference between the native (4109.69 Da) and S-alkylated peptide (4573.92 Da) allowed the assignment of eight cysteine residues linked by four disulfide bonds.

Electrospray ionization-mass spectrometry (ESI-MS) analysis of the native peptide yielded a mass spectrum with three intense 4+, 5+, and 6+ signals detected at *m*/*z* 1028.92, 823.54, and 686.28, respectively (Figure 3a). The insets shown in Figure 3a,b correspond to the experimental isotopic distribution of the [M + 6H]^6+^ ions of the native and carbamidomethylated peptides, respectively. The deconvoluted ESI-MS spectra of the native, reduced and carbamidomethylated peptides showed intense signals, corresponding to molecular masses of 4109.69 Da and 4573.92 Da (data not shown), respectively. The mass increment of 464.22 Da, after reduction and carbamidomethylation (Figure 3a,b), can be assigned to the presence of eight cysteine residues, linked by four S–S bonds, in the I1_xm11a peptide.

### 2.5. Identification of the Conotoxin in the Transcriptome Database Using a Sequence Tag Extracted from the MS/MS Spectrum

**Figure 4:**
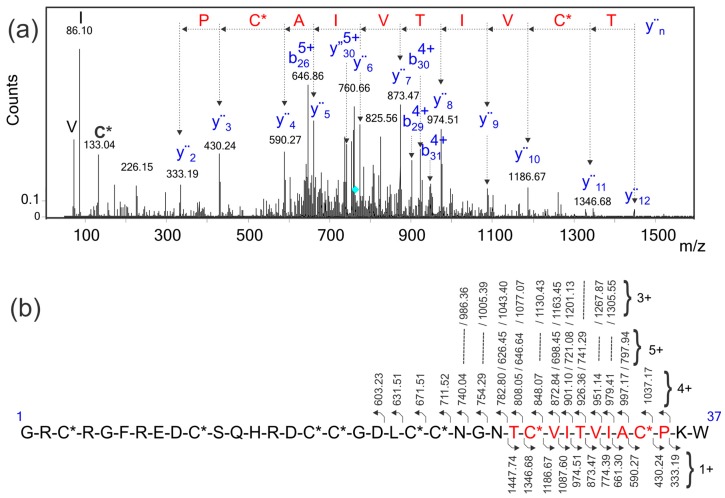
(**a**) Manual interpretation of the ESI-MS/MS spectrum of the [M + 6H]^6+^ precursor ion (*m*/*z* 763.32) of the reduced-S-alkylated conotoxin, I1_xm11a. The cyan rhombus indicates the precursor ion selected for fragmentation by CID. The sequence tag, (333.19, PC*AIVTIV C*T, 1447.74), written in red was extracted by considering the singly-charged y″_n_ ions, and was used to identify the conotoxin of interest in the transcriptome database; (**b**) Assignment for the backbone y″_n_ and b_n_ fragment ions observed in this MS/MS spectrum. The eight cysteine residues are carbamidomethylated and are represented as C*. Four expanded ranges of this MS/MS spectrum, as well as the assignment of fragment ions, are shown in Figure S2.

In order to identify the conotoxin of interest in the venom gland transcriptome database, multiply-charged ions (Figure 4a) of the reduced and S-alkylated peptide were fragmented using CID. The MS/MS spectra were exported as mgf files; these files were evaluated using three different software products (MASCOT [25], Protein Pilot [26], and Peaks [21]) developed for protein identification in sequence databases composed of the transcriptome of the venom gland of *C. ximenes*. Unfortunately, no sequence identification was retrieved with a significant score. The MS/MS spectrum of the [M + 6H]^6+^ precursor ion (Figure 4a, *m*/*z* 763.32) was manually analyzed and a reliable sequence tag of ten amino acids was extracted, considering singly-charged y″_n_ ions (333.19, PC*A-L/I-VT-L/I-VC*T, 1447.74, C*: Carbamidomethyl cysteine and L/I: Leucine/isoleucine were not differentiated). This partial sequence was also present in the MS/MS spectra of 3+, 4+, and 5+ precursor ions of the same peptide (data not shown). This information allowed the identification of a unique conotoxin in the transcriptome database, which satisfies this sequence tag [22], considering the y″_n_ ions and containing the eight previously assigned Cys residues (Figure 3). The calculated (4109.69 Da) and experimental (4109.70 Da) molecular masses for this native conotoxin, that has 37 amino acids, corresponded within a mass error of 2.4 ppm.

### 2.6. Sequence Verification of the I1_xm11a Toxin

In order to verify this identification, the reduced and S-alkylated I1_xm11a was digested with trypsin and analyzed via LC-MS/MS. Only one tryptic fragment of I1_xm11a (15–36, *m*/*z* 863.02, 3+ and *m*/*z* 1294.02, 2+, see Figure S3) was identified in the transcriptome sequence database, with a significant score above 1% of the false discovery rate (FDR) using MASCOT, Protein Pilot, and Peaks. The sequencing of this tryptic peptide only allowed the verification of the C-terminal 22 amino acids, containing six out of the eight Cys residues expected in the identified I1_xm11a conotoxin (Figure 3 and Figure 4b). Additionally, this peptide (15–36), I1_xm11a, contains the sequence tag used for protein identification (10 amino acids highlighted in red in Figure 4a,b).

The EThcD spectrum obtained for the [M + 6H]^6+^ parent ion (*m*/*z* 685.95, see Figure S4) of the native toxin showed the fragmentation of one (Cys^3^–Cys^34^) out of the four disulfide bonds [10,11,27], and also confirmed six previously-assigned C-terminal amino acids (Figure 4a,b and Figure S3). The molecular masses of the two N-terminal ions, assigned as a_6_^2+^ and b_9_^2+^, were in agreement with the identified conotoxin.

**Figure 5:**
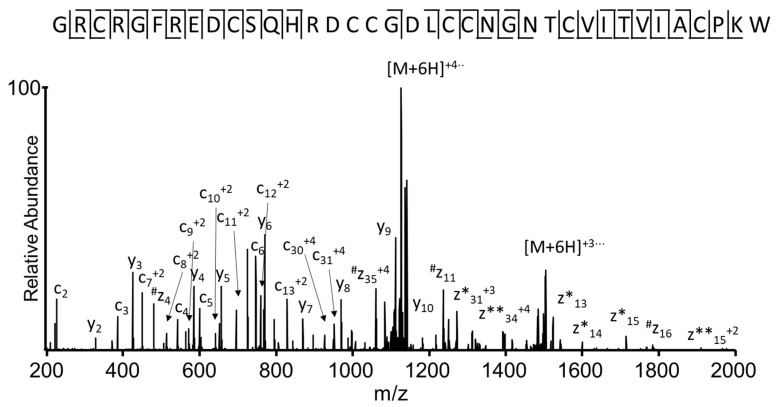
Electron transfer higher energy collision dissociation (EThcD) spectrum, recorded on the +6 precursor (*m*/*z* 763.32) of peptide I1_xm11a, after reduction and alkylation of cysteine residues, using iodoacetamide. N-terminal fragment; c ions are indicated by ⌉ and C-terminal fragment, y and z·ions are indicated by L. Multiply-charged fragment ions are indicated with the corresponding charge state z·ions resulting from cleavage at cysteine, and loss of the cysteine side chain is indicated with #. Charge-reduced species are labeled in the spectrum with ●, indicating the number of electrons transferred to the precursor ion. z ions with extra protons are indicated by *, where the number of * represents the number of protons added to the z ion.

The EThcD spectrum of the reduced and S-alkylated peptide (Figure 5) increased the sequence coverage of the identified conotoxin, providing the sequencing of thirteen N-terminal amino acids (^1^G-H^13^). Additionally, this EThcD spectrum (Figure 5) allowed automatic identification of the conotoxin in the transcriptome venom gland sequence database. The mass difference between the *m*/*z* value for the experimental (*m*/*z* 822.9456) and expected [M + 5H]^5+^ ion mass corresponds to 0.97 ppm.

### 2.7. Sequence Analysis of the Identified Conotoxin

A BLAST search revealed that this conopeptide, isolated from *C. ximenes*, belongs to the I1 superfamily [28], and its cysteine residues are arranged according to the XI framework (C–C–CC–CC–C–C) reported by Jimenez et al. [29]. Thus, the naming of this conotoxin, I1_xm11a, was in agreement with the nomenclature established for peptides that have been isolated from cone snail venom [30]. Briefly, lowercase letters assign the species “xm”; an Arabic number represents the cysteine framework “11”, and the order of discovery is indicated by a lowercase letter, starting with “a”. Furthermore, [31] has recently proposed incorporating the superfamily (I1) into the conotoxin superfamily group, to which the conotoxin I1_xm11a belongs. This is the first conotoxin described for *C. ximenes*.

## 3. Discussion

*M. tuberculosis* is responsible for an increasing amount of morbidity and mortality worldwide, and multidrug resistance has become a serious concern for current therapy strategies. According to these facts, the continued search for new compounds with anti-TB activity with different mechanisms of actions is fully justified. In order to find polypeptide species with anti-TB activity, Conidae venom has not yet been explored, probably because there is no direct link between these organisms.

The rationale for exploring anti-TB activity in this work is based on the great diversity of Conidae venom, and also because there are many conotoxin target membrane receptors. In the case of Mtb, several membrane receptors are involved in nutrient intake, a process that is essential for survival and colonization of the respiratory tract [2]. For these reasons, we hypothesized that some components in the venom of *C. ximenes* may have anti-TB activity.

Due to its very high resolving power, and its very easy optimization, RP-HPLC chromatography has been systematically used to fractionate complex polypeptide mixtures, such as venom, either for screening purposes or just for the purification of some components of interest [28,29,32].

The RP-HPLC profile of the venom extracted from *C. ximenes* revealed the presence of several fractions (Figure 1), in agreement with the great complexity reported for Conidea venom. This work constitutes the first analyses performed on the venom of *C. ximenes*, and, at the same time, is the first report of anti-TB activity screening in venom of Conidea species.

Our experimental antimycobacterial susceptibility assays used the H37Rv strain because it is accepted worldwide as a standard, representing the majority of drug-susceptible clinical isolates [33].

There are different methods available to evaluate the anti-tuberculosis activity of natural products, and no specific concentration parameters have been established for screening purposes [2,34,35]. Moreover, there is no consensus on the cut-off for the identification of potential molecules with anti-tuberculosis activity. In previous studies characterizing natural extracts, the MIC values were set to ≤100 µg/mL [34]; ≤200 µg/mL [35]; and ≤1600 µg/mL [36] for *M. tuberculosis* and other related mycobacteria strains.

In this study, we started by screening complex fractions until the chemical entity (conotoxin) responsible for anti-TB activity was determined. In order to consider a fraction with activity against Mtb, the corresponding inhibitory concentration needed to be lower than 200 µg/mL; this was the case for the fraction highlighted in gray in Figure 1. The growth-inhibitory effect in the micromolar range positioned this peptide in the same concentration order of two other drugs that are commonly prescribed for anti-TB therapeutic intervention (INH and EMB, Figure 2a).

The obtained data suggest a specific effect of I1_xm11a against a mycobacteria-tested strain, since the effect is potentiated at higher concentrations, in a dose-dependent manner (Figure 2b). This is an important parameter in pharmacology, where the concentration-dependence of antibiotics should enhance antibacterial activity via an increase in the dosage level of drugs [37]. Our results indicate that *M. tuberculosis* was slightly susceptible at low concentrations, showing at least 50 percent of inhibition of tubercle bacilli at 3 µM (*p* < 0.01, Figure 2b). The MIC of this peptide is three times more potent than that of EMB (MIC 9.8 µM, Figure S1), a drug used in current anti-TB therapy.

The low-micromolar MIC (3 µM) makes I1_xm11a conotoxin very attractive as a potential drug, and also provides the possibility of exploring its chemical space to obtain mutants with an MIC in the submicromolar range and developing more potent anti-TB drugs.

Additionally, if the biological activity of this peptide is mediated by targeting a specific receptor at the Mtb membrane, its identification deserves special attention, in order to develop a new generation of peptide-based drugs.

The sequencing of the venom gland transcriptome was very useful for two reasons: On one hand, it allowed the construction of a sequence database that facilitated the unequivocal identification of the conotoxin of interest, using the MS/MS and EThcD spectra of the peptide of interest, fragmented using CID and EThcD, respectively. Additionally, the assignment of this peptide was confirmed by digestion with trypsin and sequencing using MS/MS of a C-terminal fragment (15–36) and its identification in the transcriptome sequence database.

On the other hand, transcriptome analyses allowed us to retrieve the signal peptide, which is very important in conotoxin classification. Conotoxins are highly different from one another in terms of structure and function; however, they are all translated from mRNA as a prepropeptide, where the signal peptide is highly conserved in groups of toxins. Thus, according to their evolutionary relationships, specifically in regards to the identity between their signal peptides, conotoxins have been classified as “gene superfamilies” [38].

The members of the I1 superfamily, which have been fully characterized, are well-known for their excitatory activities [29], specifically, for their affinity for voltage-gated Na^+^ channels [39,40]. Although BLAST sequence alignment allowed the classification of I1_xm11a into the I1 superfamily, it is worth mentioning that the toxin region only presented similarities to members that have not been characterized (Figure 5).

**Figure 6:**
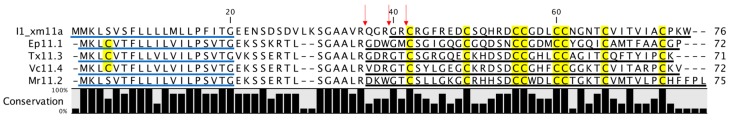
Alignment for I1_xm11a with similar conopeptides belonging to the I1-superfamily, and with framework XI. Ep11.1 corresponds to a conotoxin from *Conus episcopatus*; Tx11.3, from *Conus textile*; Vc11.4, from *Conus victoriae*; and Mr11.2, from *Conus marmoreus*. Signal peptides are underlined in blue, while mature peptides are underlined in black. Cys residues are highlighted in yellow and a conservation percentage for each amino acid is also presented in bar graph format at the bottom of the figure. The arrows in red indicate the three possible sites for N-terminal processing, according to the mechanism proposed by Dutertre et al. [6].

As we can see in Figure 5, the length of the mature I1_xm11a toxin differs from the lengths predicted for the aligned toxins: Ep11.1, Tx11.3, Vc11.4, and Mr11.2. The mature conotoxin homologs were only discovered at a cDNA level; hence, the mature toxin region was predicted based on R/K cleavage sites and via similarities to other conopeptides. Furthermore, it is known that the amount of conotoxin mRNA being expressed in a venom duct is much lower (approx. hundreds) than the number of conopeptides found (approx. thousands). Dutertre et al. [6] proposed a mechanism called “Variable Peptide Processing” (VPP), which can contribute to this exceptional diversity of conopeptides in Conidae venom. The VPP mechanism proposes that conopeptides can be differentially processed, in both their N- and C-terminals. The variants correspond to enzyme processing at alternative R/K cleavage sites in the sequence, which, in the case of I1_xm11a, could be at three possible R cleavage sites (see arrows in Figure 6).

The divergence, based on the sequence homology of I1_xm11a with members of the I1-superfamily (those that have been describe to date), suggests that our peptide presents a different molecular target for the voltage-gated Na^+^ channels in cell membranes. Previous analyses of natural products led to the identification of biological extracts from marine sources with anti-tuberculosis activities [2]; however, few studies achieved the isolation of a single molecule. According to the activity of all conotoxins reported, there is a high possibility that the activity of I1_xm11a is being targeted in a receptor or a channel in the cell membrane of Mtb.

Even though the combination of different techniques allowed the retrieval, without any doubt, of the whole sequence of the conotoxin, it is worth mentioning that the certainty of the pharmacological determination depends on the future successful production of the recombinant or synthetic peptide.

Although this hypothesis for the receptor or ion channel as a target protein must be demonstrated or refuted, knowledge regarding the various influx and efflux pathways in cell envelopes of mycobacteria, has been of interest in the design of new molecular targets for chemotherapeutic combinations against *M. tuberculosis*. The limitations of current TB treatments have driven the goals of shortening and simplifying treatment. The therapeutic dose of first-line drugs, such as isoniazid and ethambutol, needs to be sustained for several months.

In order to expand the options of molecular targets with antimycobacterial activity, ion channels or membrane receptors involved in the equilibrium of pathogens, can be of great help in future trials.

Otherwise, I1_xm11a, with its anti-TB potential, opens up the question of whether other conotoxins can also be isolated from the venom of other members of Conidae family with anti-TB activity.

## 4. Conclusions

In this work, we reported on a new molecular entity (a conopeptide named I1_xm11a), isolated from the venom gland of *Conasprella ximenes*; our data demonstrate its antimycobacterial activity in the low micromolar range (3 µM). The MIC of this conotoxin is comparable to other leading drugs that are currently in use for TB treatments, especially that of EMB.

Additionally, the work presented here shows, for the first time, the potentialities of Conidea venom as a pharmacological tool against Mtb. Sequence homology suggested that other conopeptides reported for the Conidae family might also have antimycobacterial activities, indicating that this property has been underexplored and new efforts in this direction deserve attention, due to the increasing problem of drug resistance developed by Mtb under current therapeutic treatments.

## 5. Materials and Methods

### 5.1. Specimens Collection and Venom Purification

Specimens of *Conasprella ximenes*, a mollusk-hunting cone snail [41], were collected from the sandy sublittoral area of Bahía de Los Angeles, Ensenada, Baja California, Mexico. The organisms were identified by means of mitochondrial 16S sequence. The venom ducts of 20 specimens of *C. ximenes* were dissected and immediately homogenized on ice, in 1 mL of 40% (*v*/*v*) acetonitrile (ACN) (Fermont) containing 0.1% (*v*/*v*) trifluoroacetic acid (TFA) (Fluka). The homogenate was centrifuged at 10,000× *g* for 10 min at 4 °C and the supernatant was lyophilized and stored at −80 °C until peptide purification.

### 5.2. Peptide Purification

The solubilized venom was fractionated by means of reverse phase-high performance liquid chromatography (RP-HPLC) (Agilent 1220 Series LC System), with an analytical C18 Zorbax 300SB column (4.6 × 250 mm, 5-μm particle size) and a Zorbax 300SB C18 pre-column (4.6 × 12.5 mm, 5-μm particle size), previously equilibrated in a solution of 0.12% (*v*/*v*) TFA (Solution A). The total venom retained in the pre-column was desalted with the same equilibration solution at a flow of 1.0 mL/min over 5 min. Fractions of the venom components were collected every 5 min and were eluted with a linear gradient from 0% to 60% (*v*/*v*) of pure ACN containing 0.10% (*v*/*v*) of TFA (Solution B), over 65 min, at a flow rate of 1.0 mL/min. Each fraction was tested in a cell growth inhibition assay. The chromatographic fraction containing the peptide of interest was repurified, via RP-HPLC, using a linear gradient, from 22% to 45% (*v*/*v*) of Solution B, over 60 min, at a flow rate of 1.0 mL/min. All purification steps were conducted at room temperature and the absorbance was monitored at 230 nm. The purified peptide was lyophilized, quantified by weight, and named “I1_xm11a” (nomenclature is explained in Section 3). Deionized water was purified using a Milli-Q system (Pure Lab Flex, Elga from Ion Torrent, Life Technologies).

### 5.3. Antimycobacterial Susceptibility Assay

Antimycobacterial testing was performed using a colorimetric CellTiter 96^®^ AQueous One Solution Cell Proliferation Assay (Promega, Madison, WI, USA). The test was performed in 96-well sterile microplates. All wells received 100 µL of Middlebrook 7H9 Broth (Becton Dickinson, Franklin Lakes, NJ, USA), supplemented with 0.2% (*v*/*v*) glycerol (Sigma-Aldrich, St. Louis, MO, USA), and 10% (*v*/*v*) oleic acid, albumin, dextrose and catalase (OADC, Edmond, OK, USA; Becton Dickinson, Franklin Lakes, NJ, USA). The strain H37Rv (ATCC 27294), was used as the reference-susceptible strain. The inoculum was prepared from fresh Lowenstein Jensen medium and was resuspended in Middlebrook 7H9 Broth. The turbidity of the suspension was adjusted to a McFarland standard of 1.0. The suspension was homogenized and allowed to precipitate larger particles. The supernatant was diluted at a ratio of 1:20, and 100 µL was used as inoculum. To reduce evaporation from the plates, 200 µL of sterile water was added to all outer perimeter wells. Each microplate was incubated for 7 days at 37 °C. Following incubation, 10 µL of CellTiter 96^®^ solution (158 µg/mL) was added to each well. The plates were re-incubated at 37 °C for two to four hours. The absorbance, recorded at 450 nm using an Epoch Microplate Spectrophotometer, was used to indicate bacterial growth. In order to know the maximal growth reference, growth controls (GC) with 100 µL of inoculum plus 100 µL of medium were used. Drugs to treat tuberculosis, such as INH and EMB, were considered positive controls.

In the first screening, several fractions from *Conasprella ximenes* venom were evaluated at 200 µg/mL each. The peak that contains the conotoxin of interest (later named I1_xm11a) with antimycobacterial susceptibility was tested at 100 µg/mL (24 µM), and its minimal inhibitory concentration (MIC) was determined, as described below.

### 5.4. Minimal Inhibitory Concentration Assay

The MIC of I1_xm11a was tested in 96-well sterile microplates. All wells received 100 μL of supplemented Middlebrook 7H9 broth. One-hundred microliters of a 4× working solution of I1_xm11a were added to the first row of each column. One-hundred microliters were transferred from row 1 to row 2, and the contents of the wells were homogenized by pipetting. Identical serial 1:2 dilutions were continued through the rows, and 100 μL of excess medium was discarded from the well in the final row. Subsequently, 100 μL of *M. tuberculosis* inoculum was added to the wells. The final test concentration range was 24–0.75 µM. The concentration ranges for the positive controls (for INH and EMB) were also evaluated. The MIC was defined as the lowest concentration of I1_xm11a with a statistical difference versus growth control. Each experiment was performed in triplicate.

### 5.5. Reduction and S-Alkylation

Approximately 1 µg of purified I1_xm11a was dissolved in 10 μL of 50 mM ammonium bicarbonate buffer (containing 0.5 mol/L of guanidinium chloride pH 8.3). Dithiothreitol (DTT) was added, at a final concentration of 5 mM, and the solution was incubated for 1 h at 37 °C. After the reaction reached room temperature, an aqueous solution of iodoacetamide (IAM) was added to reach a final concentration of 10 mM. The reaction proceeded for an additional 30 min in darkness at room temperature. The reaction was acidified by adding 1 μL of 10% (*v*/*v*) formic acid (FA) and immediately analyzed by LC-MS/MS. All reagents were obtained from Sigma-Aldrich.

### 5.6. LC-MS/MS Analyses

The native peptide and the S-alkylated peptide were loaded into a 40-nL enrichment precolumn, packed with 5-μm particles (300SB-C18) and 300 Å (ProtID-Chip 43 II, Agilent Technologies, Santa Clara, CA, USA), which was previously equilibrated with 0.1% (*v*/*v*) TFA solution. The samples were desalted by passing five volumes of the precolumn with the same equilibrium buffer at 2 μL/min. After finishing the desalting step, the precolumn was coupled online to the analytical reversed phase column (75 mm, 43 μm), filled with the same packing material previously mentioned. The peptides were eluted at a constant flow rate of 400 nL/min in a linear gradient from 3% to 70% of a solution containing 90% of ACN/0.1% of FA, for 15 min. Afterwards, the peptides were sprayed into a G6530AA Accurate Mass quadrupole time-of-flight (QTOF) LC/MS System (Agilent Technologies, Santa Clara, CA, USA), using 1900 V and 65 V as the capillary and skimmer voltages, respectively. The fragmentor was set at 175 V. The high-resolution mass spectra (m/∆m~20,000) were acquired from *m*/*z* 300–2000 Da at 2 GHz, in MS mode to detect the molecular mass of the peptides of interest. Collision energies (CE) were customized to obtain an efficient fragmentation of the 4+, 5+, and 6+ ions, derived from the reduced and S-alkylated peptides. The data were acquired in positive ion mode and were analyzed using MassHunter Qualitative analysis software (B.06.00) from Agilent Technologies. The resultant ESI-MS/MS spectra were manually interpreted to extract a partial sequence.

The native, or the reduced and S-alkylated I1_xm11a peptides were also analyzed in an Acclaim PepMap100 C18 75 μm × 2 cm column with a 3-μm bead size, coupled to an EASY-Spray 75 μm × 50 cm PepMap C18 analytical HPLC column with a 2-μm bead size, using the auto sampler of an EASY-nLC 1200 HPLC. Toxin was eluted with a linear gradient of 0–45% B for 30 min, 45–56% of B for 10 min, and 56–100% B for 10 min (A = 2% acetonitrile in 0.5% acetic acid, B = 90% acetonitrile in 0.5% acetic acid), using the EASY-nLC 1200 HPLC in an Orbitrap Fusion Lumos mass spectrometer. High resolution full MS spectra were acquired at a resolution of m/Δm 60,000, automatic gain control (AGC, Walloon Brabant, Belgium) target of 4e5, maximum ion time of 50 ms, and scan range of 300–1400 *m*/*z*. To obtain a high-quality spectrum, two charge states of the native toxin (*m*/*z* = 822.945 (+5) and 685.955 (+6)) and two charge states of the alkylated toxin (*m*/*z* = 915.7916 (+5) and 763.328 (+6)) were targeted. For the native toxin, EThcD was used for fragmentation with an ETD reaction time of 10 ms, ETD reagent ion AGC of 2e5, EThcD as supplemental activation with normalized collision energy of 35%, a resolution of 15,000, an AGC target value of 4e5, and 10 microscans per spectrum. For the alkylated toxin, EThcD was used for fragmentation, with an ETD reaction time of 30 ms, ETD AGC setting of 2e5, EThcD as supplemental activation with normalized collision energy of 30%, a resolution of m/Δm 30,000, maximum injection time of 150 ms, and AGC target value of 5e4. The data were searched against the in-house generated *Conasprella ximenes* database using Byonic [42]. To maximize sequence coverage, the MS/MS data of the same precursor were averaged over the entire elution time of the toxin.

### 5.7. Transcriptome of the Venom Gland

Venom glands of six *C. ximenes* specimens were dissected under RNAse-free conditions and pooled in a single tube. RNA was extracted using the SV Total RNA Isolation System (Promega, Madison, WI, USA). After assessing RNA quality and quantity through the absorbance ratio of 260/280 nm (1.8–2.0), RNA integrity was reconfirmed using a 2100 Bioanalyzer instrument (Agilent Technologies, Santa Clara, CA, USA).

cDNA library preparation and sequencing were performed at the Core Facility of the Institute of Biotechnology (Cuernavaca, Mexico). The cDNA library was generated using an Illumina platform (Genome Analyzer IIx) with a TruSeq Stranded mRNA Sample Preparation Kit. A 72-base pair (bp) paired-end sequencing scheme was chosen, with cDNA fragments ranging in size from 200–400 bp. The reads obtained were clipped-off from their adaptor sequences and were followed by an assessment of the quality of the cleaned raw reads using FastQC [43].

In order to assemble good quality single reads into the contiguous consensus sequences (contigs), Trinity (v.2.0.2) software was used [44]. Transcriptome assembly was performed using the following settings: Trinity –seqType fq –max_memory 60 G –min_contig_lenght 100 –normalize_max_read_cov 30 –left R1.fastq –right R2.fastq –SS_lib_type RF –CPU 27 –no_bowtie [45]. A database of open reading frames longer than 50 amino acids was generated from the transcriptome assembly, using the Transdecoder utility included in Trinity [44]. Finally, redundant sequences were removed from the dataset. The transcriptome sequence database is available upon request to Alexei Licea-Navarro (corresponding author).

### 5.8. Database Search

The MS/MS spectra were exported into an mgf file format and loaded by three database search engines (MASCOT [25], Protein Pilot [26] and Peaks [21]) to identify the conotoxin of interest in the venom gland transcriptome database assembled for *C. ximenes*. In all software packages, the mass tolerance for matching the precursor and fragment ions were set to 0.1 and 0.05 Da, respectively.

No protease specificity was considered for the identification of the reduced and S-alkylated conotoxin. Carbamidomethyl cysteine was defined as a fixed modification in all experiments. In the case of Protein Pilot and Peaks, the database search considered the entire set of PTMs, defined in their libraries, which contain all modified amino acids frequently found in conopeptides [8]. In the case of MASCOT, several combinations of four variable PTMs were explored, to avoid crashing the software.

Manual interpretation of the MS/MS spectra were conducted to extract a reliable sequence tag that was confirmed using the b_n_ and y″_n_ backbone fragment ions. The mass accuracy (0.01–0.02 Da), as well as other aspects used in the de novo sequencing of peptides were also considered to extract this partial sequence [46,47]. The conotoxin of interest was identified in the transcriptome database with the aid of MASCOT [25], using the sequence query option. The sequence tag was searched, allowing it to be either an N-terminal or C-terminal ion series.

Identity and similarity assessments were performed using the Basic Local Alignment Search Tool (BLAST) [48], using the non-redundant database. The signal peptide sequence was calculated by means of the SignalP 4.1 server [49], while the mature peptide region was determined using the total molecular weight of the conotoxin.

## Supplementary Materials

The following are available online at http://www.mdpi.com/2072-6651/10/2/51/s1, Figure S1: MIC determination of Ethambutol and Isoniazid with pathogenic M. tuberculosis H37Rv strain, Figure S2: ESI-MS/MS spectrum of I1_xm11a toxin, Figure S3: ESI-MS spectrum of the tryptic peptide (15-36) derived from the reduced and S-alkylated conotoxin I1_xm11a, Figure S4: EThcD of native peptide I1_xm11a spectrum.
