# Supplementary material for: Antimycobacterial Activity: A New Pharmacological Target for Conotoxins Found in the First Reported Conotoxin from Conasprella ximenes
Source: Toxins (Basel). 2018 Jan 23;10(2):51. doi: 10.3390/toxins10020051 (PMC5848152; doi:10.3390/toxins10020051)
Supplement: Supplementary file 1 [file toxins-10-00051-s001.pdf]

Supplementary Materials: Antimycobacterial Activity: A New Pharmacological Target for Conotoxins Found in the First Reported Conotoxin from *Conasprella ximenes*

Andrea Figueroa-Montiel, Johanna Bernáldez, Samanta Jiménez, Beatrix Ueberhide, Luis Javier González and Alexei Licea-Navarro

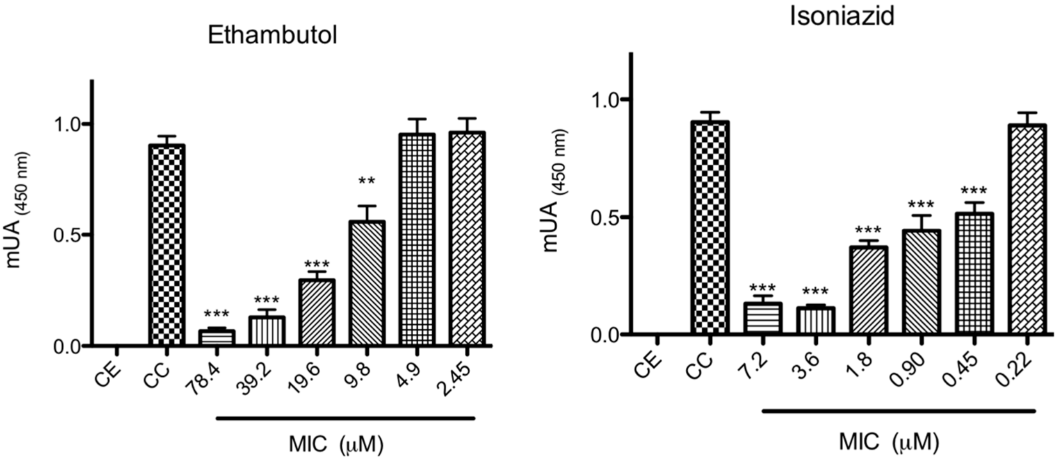

Figure S1. MIC determination of Ethambutol and Isoniazid with pathogenic *M. tuberculosis* H37Rv strain. MIC for EMB and INH, were 9.8 and 0. 45 μM respectively. The statistical significance of differences between treatments and growth control was analyzed by Student´s t-test. **P<0.01, ***P<0.001 vs. Growth Control (GC). Experimental control (EC) correspond to sterility media without inoculum

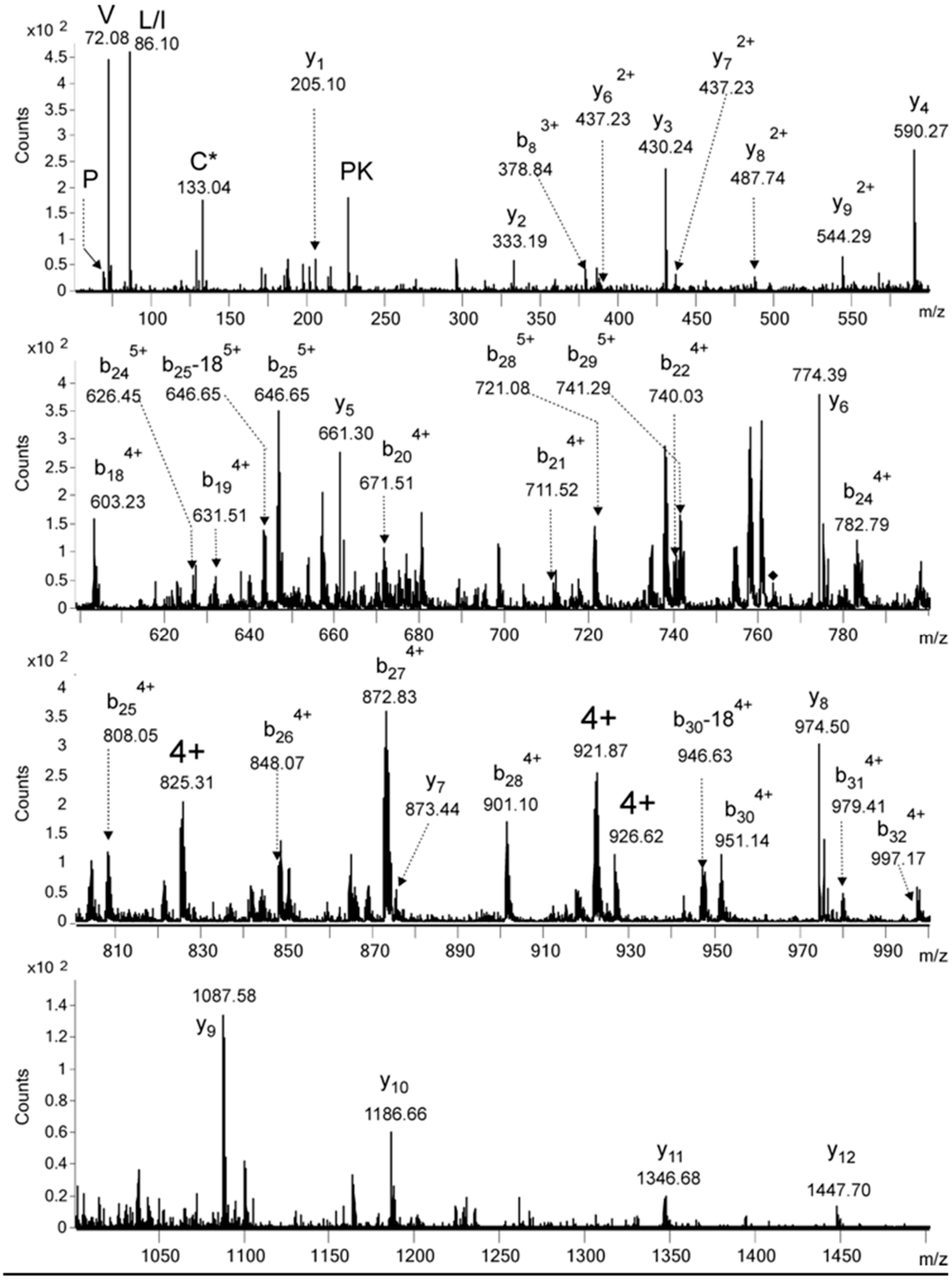

Figure S2. ESI-MS/MS spectrum of I1_xm11a toxin. Four expanded ranges of the ESI-MS/MS spectrum of the $[M+6H]^{6+}$ ion derived from the reduced and S-alkylated I1_xm11a toxin detected at m/z 763.32 with the assignment of the fragment ions.

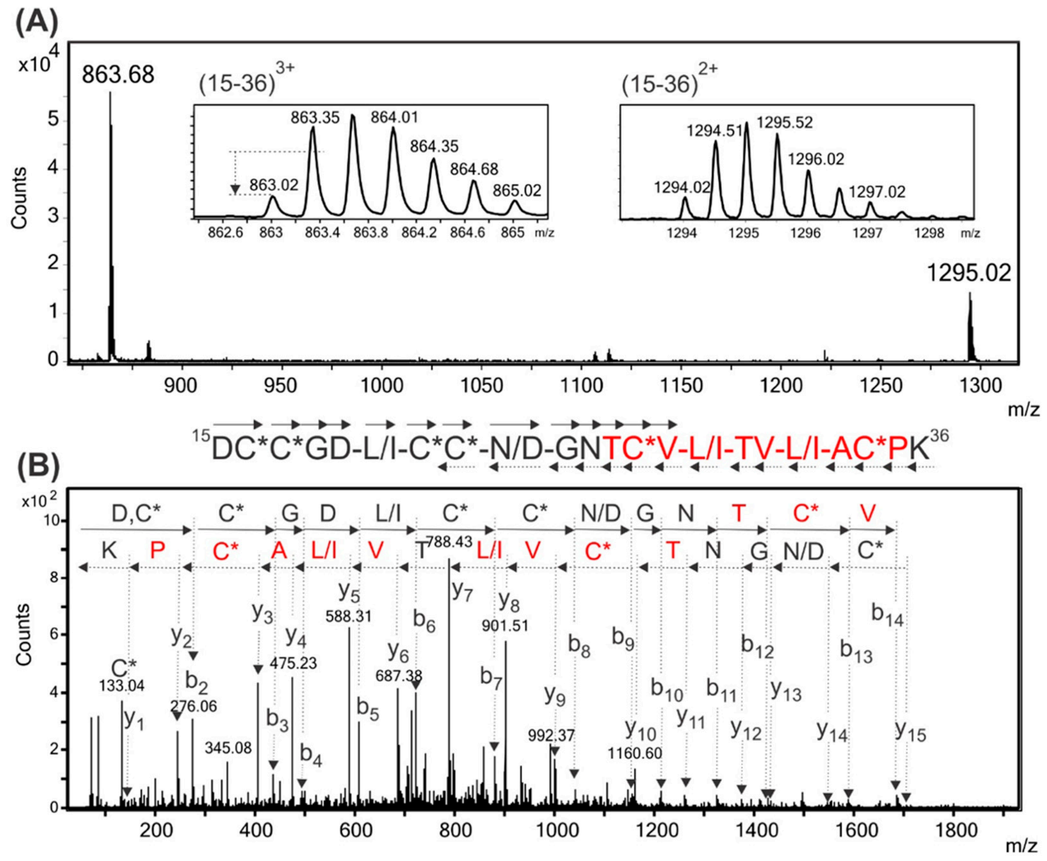

Figure S3. ESI-MS spectrum of the tryptic peptide (15-36) derived from the reduced and S-alkylated conotoxin I1_xm11a. The insets in **(A)** contains expanded regions of this ESI-MS spectra showing the isotopic ion distributions of the (15-36)2+ and (15-36)3+ ions. The broken lines in the left inset shows the decreased intensity observed for the monoisotopic ion detected at m/z 863.02 probably due to a partial deamidation process (N/D) of Asn23during digestion. The ESI-MS/MS shown in **(B)** correspond to triply-charged ion detected in **(A)** at m/z 863.02. The sequence highlighted in red correspond to the partial sequence used in the manuscript to identify the conotoxin in the venom gland transcriptome sequence database.

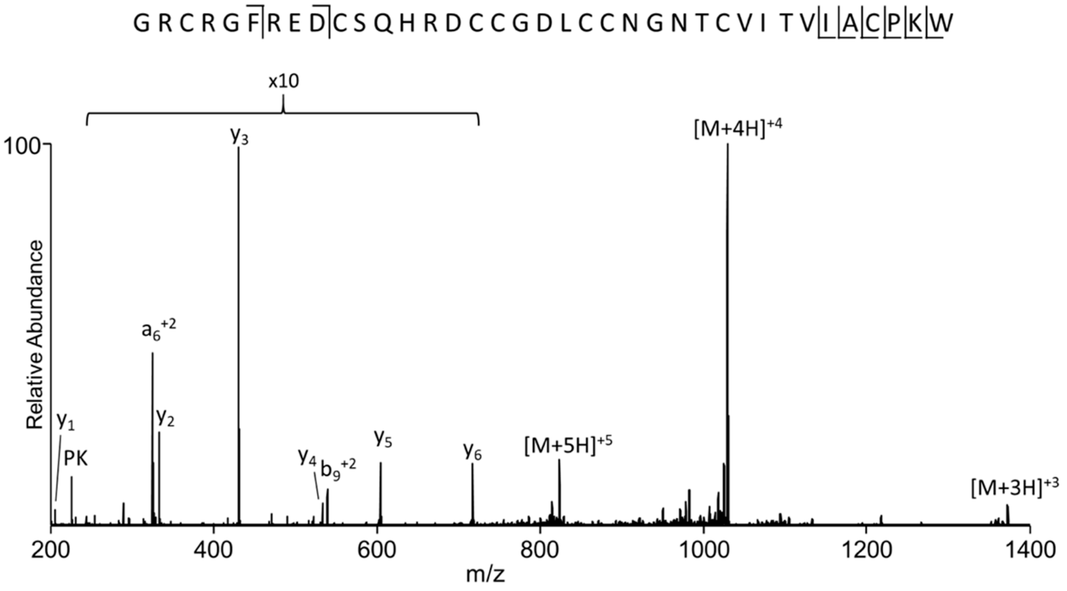

Figure S4. EThcD of native peptide I1_xm11a spectrum. EThcD spectrum recorded on the 6+ precursor (m/z 685.95) of the native disulfide bonded peptide I1_xm11a. N-terminal fragment ions (a and b) are indicated by] and C-terminal fragment ions (y ions·) are indicated by L. Multiply charged fragment ions are indicated with the corresponding charge state. Precursor ions and internal fragment ions are labeled in the spectrum.
